# Vibrotactile Feedback for Improving Standing Balance

**DOI:** 10.3389/fbioe.2020.00094

**Published:** 2020-02-21

**Authors:** Giulia Ballardini, Valeria Florio, Andrea Canessa, Giorgio Carlini, Pietro Morasso, Maura Casadio

**Affiliations:** ^1^Department of Informatics, Bioengineering, Robotics and Systems Engineering, University of Genoa, Genoa, Italy; ^2^Department of Robotics, Brain and Cognitive Sciences, Italian Institute of Technology, Genoa, Italy

**Keywords:** somatosensory integration, vibrotactile feedback, postural control, biofeedback, balance

## Abstract

Maintaining balance standing upright is an active process that complements the stabilizing properties of muscle stiffness with feedback control driven by independent sensory channels: proprioceptive, visual, and vestibular. Considering that the contribution of these channels is additive, we investigated to what extent providing an additional channel, based on vibrotactile stimulation, may improve balance control. This study focused only on healthy young participants for evaluating the effects of different encoding methods and the importance of the informational content. We built a device that provides a vibrotactile feedback using two vibration motors placed on the anterior and posterior part of the body, at the L5 level. The vibration was synchronized with an accelerometric measurement encoding a combination of the position and acceleration of the body center of mass in the anterior-posterior direction. The goal was to investigate the efficacy of the information encoded by this feedback in modifying postural patterns, comparing, in particular, two different encoding methods: vibration *always on* and vibration with a *dead zone*, i.e., silent in a region around the natural stance posture. We also studied if after the exposure, the participants modified their normal oscillation patterns, i.e., if there were after effects. Finally, we investigated if these effects depended on the informational content of the feedback, introducing trials with vibration unrelated to the actual postural oscillations (*sham* feedback). Twenty-four participants were asked to stand still with their eyes closed, alternating trials with and without vibrotactile feedback: nine were tested with vibration *always on* and *sham* feedback, fifteen with *dead zone* feedback. The results show that synchronized vibrotactile feedback reduces significantly the sway amplitude while increasing the frequency in anterior-posterior and medial-lateral directions. The two encoding methods had no different effects of reducing the amount of postural sway during exposure to vibration, however only the *dead-zone* feedback led to short-term after effects. The presence of *sham* vibration, instead, increased the sway amplitude, highlighting the importance of the encoded information.

## Introduction

Postural control is a complex sensorimotor skill with two main functions: stabilizing balance and maintaining the relative position of body segments (Massion, 1994; Ivanenko and Gurfinkel, 2018). It requires the interaction of the sensory, muscular, and nervous systems (Horak and Macpherson, 1996). In particular, the central nervous system must process and integrate concurrent feedback from the vestibular, somatosensory, and visual sensory channels (Hirabayashi and Iwasaki, 1995; Horak and Macpherson, 1996). If those are impaired or absent, postural control and balance are compromised, increasing also the risk of falling (Maki, 1989; Brown et al., 1999; Melzer et al., 2004; Horak, 2006). These impairing sensory deficits could be caused by aging (Peterka and Black, 1989; Melzer et al., 2004), diabetes (Najafi et al., 2010), vestibular disorder or neurodegenerative diseases, such as Parkinson (Mancini et al., 2011, 2012; Marchesi et al., 2019).

Each sensory system contributes differently to postural control; thus the impairment of a specific sense has different impacts on balance. For example, during quiet standing, the postural sway increases more when somatosensory information is unavailable (Nashner et al., 1982) than in absence of the vestibular or visual information (Peterka and Black, 1989; Macpherson and Inglis, 1993; Dozza et al., 2007). In any case, the contribution of feedback from different modalities is known to be additive, thus it seems worth investigating to what extent providing an additional channel may further improve balance and/or compensate for balance deficits in pathological conditions. Several studies suggest indeed that, in presence of sensory deficits, providing a supplemental sensory information to the central nervous system might improve postural stability, decreasing the postural sway and even the risk of falling (Wall et al., 2001; Dozza et al., 2005; Danilov et al., 2007; Sienko et al., 2012). This supplemental information could play a crucial role for subjects using exoskeletons (Muijzer-Witteveen et al., 2017) or lower limb prosthetics (Lee et al., 2007), where the loss of somatosensation associated with the lesion or the amputation is an obstacle for achieving stable and efficient standing balance and walking patterns.

For those reasons, many research groups have developed devices able to provide supplemental information through biofeedback. Different sensory stimuli, such as vibrotactile (Alahakone and Senanayake, 2009; Sienko et al., 2012), electro-tactile (Tyler et al., 2003; Lee et al., 2007), visual (Alahakone and Senanayake, 2010; Nitz et al., 2010; Halická et al., 2014), auditory (Chiari et al., 2005; Dozza et al., 2005; Giansanti et al., 2009; Franco et al., 2012), or multimodal (Verhoeff et al., 2009; Bechly et al., 2013), have been used and investigated for improving postural control. In particular, vibrotactile feedback is widely used because it can provide additional information without interfering with basic functions like hearing or seeing (Haggerty et al., 2012). Usually, the vibrotactile devices use arrays of several vibration motors to convey postural sway information mainly on the torso (Van Erp, 2005; Verhoeff et al., 2009; Lee et al., 2012; Sienko et al., 2012; Xu et al., 2017). However, the feedback provided by the most common vibrotactile devices is difficult to interpret and integrate in the neural control (Culbertson et al., 2018). One reason is that the patterns of somatosensory stimuli are not intuitive or complex, due to either the number of vibration motors, thus forcing the user to process a redundant set of signals, or to the encoding methods that may require specific attention (Brewster and Brown, 2004).

While from the technological point of view there are several solutions for providing supplemental vibrotactile feedback, while which information is more effective to reduce the postural sway and how to encode it has received less attention. For example, there is evidence that humans modify their postural sway (Goodworth et al., 2009; Loughlin et al., 2011) in presence of vibrotactile feedback, encoding velocity and/or position of the body Center of Mass (CoM) or the Center of Pressure (CoP). However, other studies have shown that also a low level of vibrational noise, e.g., mechanical or electrical, is useful to improve postural stability, enhancing the sensitivity of the somatosensory system (Dhruv et al., 2002; Liu et al., 2002; Janssen et al., 2010; Magalhães and Kohn, 2011; Borel and Ribot-Ciscar, 2016; Kwak et al., 2016). This kind of stimulation (e.g., stochastic resonance) resulted in a reduction of the postural sway in elderly people (Gravelle et al., 2002; Priplata et al., 2002, 2003) and in people affected by vestibular impairments (Janssen et al., 2010). Therefore, it would be interesting to further investigate the role of the information encoded in the vibration, and the effects due to different encoding methods. In fact, for supplemental auditory or visual feedback (Dozza et al., 2006) has been demonstrated that linear and logarithmic mapping (Dozza et al., 2006) have different effects on the postural sway. Moreover, the feedback could be either continuously provided or silenced in a region around the natural stance posture, in order to avoid a sensory overload of the user (Alahakone and Senanayake, 2010).

In this framework, we designed and built a portable, low-weight and low-cost device to provide vibrotactile feedback to improve standing balance. Differently from the majority of current devices, based on arrays of vibration motors and often providing complex patterns of stimuli (Van Erp, 2005; Verhoeff et al., 2009; Lee et al., 2012; Xu et al., 2017), we used only two vibration motors placed on the opposite sides of the torso (abdomen and back) at the L5 level, namely in the CoM area. The idea was to activate them as function of the actual sway evaluated from the accelerometric signal. As explained in the Materials and Methods section the implemented system encoded in the vibrotactile feedback a combination of position and acceleration of the CoM in the sagittal plane. The main goal was to evaluate the extent such additional sensory feedback could reduce the sway amplitude. If the previous evaluation was positive, we also planned to test three related hypotheses about the improvements:

- the changes depend on the time profile of the vibrotactile stimulation, comparing a continuous stimulation paradigm with a paradigm that included a dead zone (with vibration silent) around the natural stance posture. If the continuous stimulation paradigm would not lead to better performance, the dead zone paradigm would be preferable for prolonged use of the vibrotactile feedback, because it reduces the exposure to the stimuli, avoiding the sensory overload of the user (Alahakone and Senanayake, 2010). To the best of our knowledge this hypothesis has never been tested for the vibrotactile feedback, but only for the auditory feedback. Since these two feedback channels are different, we could not assume a priori that the test would lead to similar results;
- the changes depend on the informational content of the feedback i.e., they are not a mere effect of the vibration. While there is a large amount of literature on the effects of vibration noise and about effect of arrays of vibrators, there is lack of knowledge about the mechanism of action underlying simple informative vibrotactile feedback. Specifically, in cases as the one discussed here, where feedback about postural oscillations is provided by only two vibrators, the fact that the informational content and not the vibration *per se* determines changes on the postural oscillations, was not extensively verified by previous studies.
- the proposed vibrotactile feedback do not induce after effects i.e., when the vibration is turned off the participants recovered their normal oscillations patterns, without any influence of the previously experienced vibration. In fact, after a short exposure to vibrotactile feedback, participants could immediately recover their normal oscillation patterns, or could exhibit either persistent or opposite effects with respect to the ones observed during the vibration trials. This is an important point that deserves extensive investigations, however it has received scarce attention in the literature and with this study we made a preliminary attempt to fill the gap.

To verify these hypotheses, we asked young healthy participants to stand upright with their eyes closed on a rigid horizontal surface wearing the device that included vibration motors and an accelerometer sensor. The acceleration profiles were analyzed, correlating them with the different stimulation modalities described in the Materials and Methods section.

## Materials and Methods

### Device

**Figure 1:**
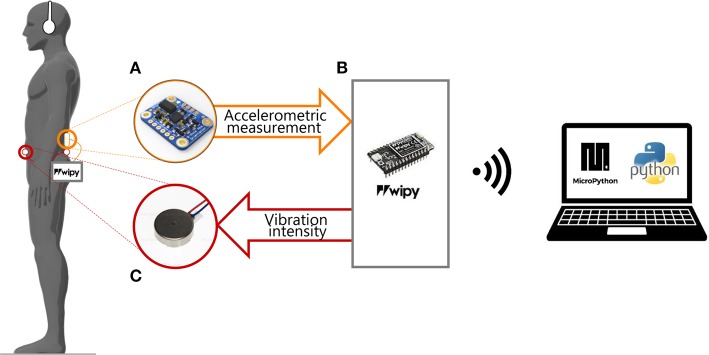
Experimental set-up. The participant was asked to stay still in the standing position, wearing headphones and our portable device composed by: **(A)** a sensor (IMU) placed on the back at L3 level; **(B)** the microprocessor unity connected to the PC via Wi-Fi; **(C)** two vibration motors attached to the skin of the participant: on the back and on the abdomen at L5 level. The IMU recorded the accelerometric signal and sent it to a microprocessor (WiPy) that saved them on a microSD card. The accelerometric measurements were used for controlling the vibration motors.

We designed a portable device that provides supplemental vibrotactile feedback synchronized with an accelerometric signal encoding information about the CoM position and acceleration. The device weights 400 g and consists of three main components: (a) an input and recording unit, based on an Inertial Measurement Unit (IMU) sensor, (b) a processing unit, and (c) a vibrotactile output unit (Figure 1).

a) Input and recording unit

The acceleration vector of the CoM is measured by means of the three-axis IMU (BST-BNO055-DS000-12, Bosch Sensortec GmbH, Germany, sensitivity = 0.2 mV = 1.2 mm/s^2^; non-linearity = 0.5 % FS, bandwidth = 62.5 Hz), firmly attached to the participants' back at the L3 level, which approximately corresponds to the CoM position during quiet standing. The accelerometer gain was preset in such a way to have a measurement in the range of 2 g, appropriate for measuring the small acceleration caused by postural adjustments. The IMU was positioned as in Mancini et al. (2011, 2012), with one of the accelerometer's axes aligned in the Anterior-Posterior (AP) direction, a second axis in the Medial-Lateral (ML) direction, and the third in the vertical direction. Thus, in correspondence of the natural equilibrium posture of each participant, the measurement signal in the AP direction has a null mean value, unaffected by any gravity component. In contrast, this component is not negligible when the body sways forward or backward with respect to the reference position, with an additional gravity component related to the tilt angle. As a consequence, the measurement signal in the accelerometer's AP direction is a combination of the CoM acceleration and the CoM position in the AP direction. The raw signal measured along the AP axis of the IMU is used as input for controlling the vibration unit (see section c) and thus the control signal used in this study encodes a combination of:

1. the component of the CoM angular acceleration along the accelerometer's AP direction, characterized by high-frequency component;
2. the projection of the gravity vector along the accelerometer's AP direction, related to the CoM position; thus, characterized by a lower frequency component.

Notice that the AP direction is considered with respect to the participants' body, thus is not parallel to the floor.

b) The processing unit

This unit is based on a microprocessor (WiPy 2.0, Pycom, Guildford) which received as input the data provided by the IMU, computed the control parameters according to the control paradigms explained in the following section, and sent the command signals to the two vibration motors. A custom-made printed circuit board connected the WiPy with the IMU and the vibration motors. The WiPy had also an ESP32 expansion board, which provided the connection to the battery (lithium-ion battery: 1 S, 1,200 mAh) and a MicroSD where were stored the accelerometric signals along the three axes. All the components of the processing unity were enclosed in a 14.5 × 7.5 × 4.5 mm module. The microprocessor communicated via WiFi with a laptop. The software of the WiPy was developed with MicroPython (Pymakr plug-in provided by Pycom).

c) Vibrotactile output unit

The AP acceleration of the CoM modulated the amplitude and frequency of the vibration provided by two micro-motors with integrated eccentric rotating mass (Pico Vibe 10 mm vibration motors; Precision Microdrives Inc., Model # 310-117). Each vibration motor had an operational frequency range of 50 to 250 Hz and peak vibrational amplitude of 2.6 g. We attached the vibration motors on the back and the abdomen of the participant, at the L5 level, i.e., distant enough from the IMU (located back at the L3 level) in order to avoid interference (Shah et al., 2019).

The vibration frequency *f* (in Hz) of each motor was computed, as a function of the control variable *a*, through a second order polynomial rule:

(1) $$\begin{array}{l} {f=\;c}_{1}{\;}^{*}|{a|}^{2}\;+\;c_{2}{\;}^{*}|a|\;+\;c_{3}; \end{array}$$

where the coefficients (c1 = −212.66, c2 = 293.34 and c3 = 150.21) were set based on:

- the minimum level of activation of these vibration motors (Krueger et al., 2017);
- the Just Noticeable Difference (JND) for this stimulus, computed according to Iandolo et al. (2019) and Shah et al. (2019).

The control variable “*a*” was related to the AP component of the accelerometric measurement (m/s^2^) as explained in the following section.

Equation 1 takes in account two components: (1) a linear relationship between the activation voltage and the acceleration signal and (2) a second order polynomial relationship between the activation voltage and the vibration frequency. The frequency and amplitude of the vibration are coupled: the frequency of vibration in Hz is roughly 100 times the amplitude in g and their relationship is linear in the range of activation (Krueger et al., 2017). Thus, controlling the frequency as in Equation 1 implies also a change of the vibration amplitude. For simplicity, in the following we refer to changes in intensity (its amplitude and frequency of the vibration) of the vibration and we express it only in terms of frequency. The reason for choosing this kind of coupled vibration motors was 2-fold: they are inexpensive and the vibration feedback is more effective when frequency and amplitude are coupled (Cipriani et al., 2012).

### Vibrotactile Feedback Control

We investigated three different methods of synchronization between the vibrotactile feedback and the accelerometric signals, namely three different encoding methods of the body sway: *Always On* (AO), *Dead Zone* (DZ), and *Sham* (S).

**Figure 2:**
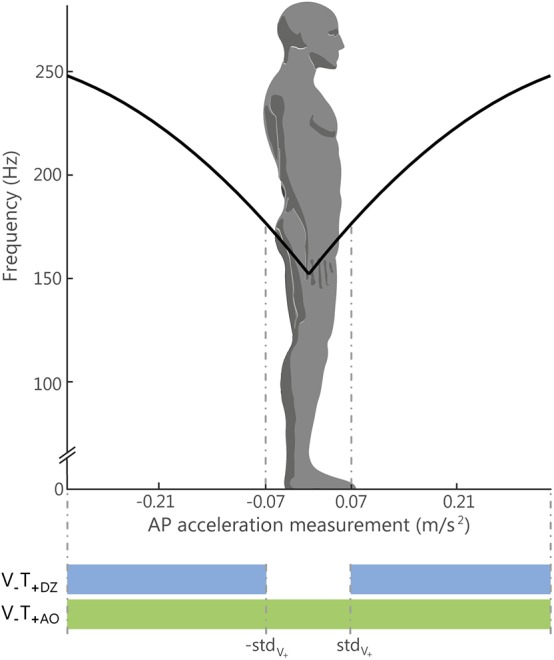
Relation between the Anterior-Posterior (AP) acceleration and the vibration frequency. The black line represents, for the always on method (V_−_T_+AO_), the relation between the amplitude of the acceleration signal measured by the IMU sensor on the AP direction in absolute unit (m/s^2^) and the vibration frequency (in Hz) applied to one motor or the other: the motor on the abdomen, for positive acceleration respect to the natural stance, and the motor on the back, for negative acceleration. The standard deviation of the acceleration measurement recorded during the initial trial with eyes open (std_V+_) was used for defining the limit of the dead zone, i.e., the region where the vibration was silent for the DZ method (V_−_T_+DZ_): this region is represented in the figure by the two dotted lines. Outside that region the vibration was controlled in in the same way for both methods (AO and DZ).

In the AO and DZ feedback methods, the control variable *a* of the vibration frequency (Equation 1) encoded the actual amplitude of the accelerometric signal along the anterior-posterior direction (*a* = *a*_*AP*_; Figure 2): the vibration motor on the back (V1) was activated when the acceleration vector was directed backward, while vibration motor on the abdomen (V2) was activated when the acceleration vector was directed forward.

More specifically, in the AO method the participants continuously felt the vibration, i.e., one of the two vibration motors was always active as explained by the following activation rule, where *c*_1_, *c*_2_, and *c*_3_ are the same coefficients of Equation 1:

(2) $$\begin{array}{l} \{\begin{array}{ccc} f_{V1}=c_{1}*{\left(\left|a_{AP}\right|\right)}^{2}+c_{2}*\left(\left|a_{AP}\right|\right)+c_{3} & f_{V2}\;=0; & a_{AP}\;<0\\ f_{V1}\;=0 & f_{V2}=c_{1}*{\left(\left|a_{AP}\right|\right)}^{2}+c_{2}*\left(\left|a_{AP}\right|\right)+c_{3} & a_{AP}\;\geq 0 \end{array} \end{array}$$

The DZ method is similar to the AO method, with the difference that vibrotactile feedback is turned off in a small region around the natural stance posture, namely if the accelerometric signal falls below a given threshold *Thr*. Thus, the activation rule is expressed by the following equation:

(3) $$\begin{array}{l} \{\begin{array}{ccc} \;f_{V1}=c_{1}*{\left(\left|a_{AP}\right|\right)}^{2}+c_{2}*\left(\left|a_{AP}\right|\right)+c_{3} & f_{V2}\;=0 & \;a_{AP}\leq \;-Thr\\ f_{V1}\;=0 & f_{V2}\;=0 & \;|a_{AP}|<Thr\\ f_{V1}\;=0 & \;f_{V2}=c_{1}*{\left(\left|a_{AP}\right|\right)}^{2}+c_{2}*\left(\left|a_{AP}\right|\right)+c_{3} & \;a_{AP}\geq Thr \end{array} \end{array}$$

The acceleration threshold was chosen to be equal to the standard deviation of the accelerometric signal recorded when the participants were standing with the eyes open during the baseline phase (see Experimental Set-Up and Protocol).

In the *sham* feedback, the vibration had the same intensity of the other two feedback methods, but did not encode any information about the actual sway of the participant. Specifically, the *sham* vibration encoded the direction and amplitude of the accelerometric signal in another trial. With this choice the vibration had the same intensity (i.e., range of frequency: 150 ÷ 235 Hz) already experienced during the other trials, but it did not encode any information about the CoM on the current trial.

### Participants

The 24 participants enrolled in the experiment were healthy young adults, who were divided in two groups. The first one was composed of 15 participants (25.13 means ± 2.19 std years, 8 females) who were tested with the DZ feedback method. The second group was composed of 9 participants (25.78 ± 3.49 years, 5 females) who were tested with the AO feedback method. The latter group was tested also with the *sham* feedback at the end of the experiment.

For both groups the inclusion criteria were the same: no known history of disease or lower limb injury, normal cognitive abilities, no problems of visual integrity that could not be corrected with glasses or contact lenses.

All participants provided written consent to participate in this experiment. The study was conformed to the standard of the declaration of Helsinki and was approved by the institutional ethical committee (Comitato etico regione Liguria).

### Experimental Set-Up and Protocol

**Figure 3:**
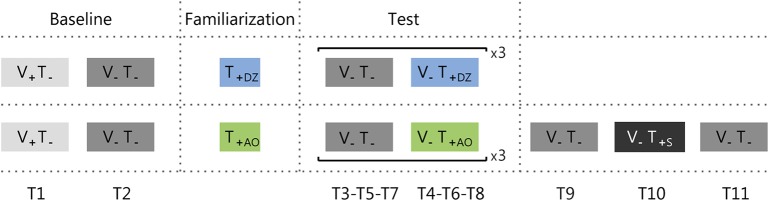
Protocol adopted for group 1 (upper row) and group 2 (bottom row). Trials were either with the visual feedback (i.e., eyes open: V_+_), or without it (i.e., eyes close: V_−_). The vibrotactile feedback was either off (T_−_), or on (T_+_). There were three types of vibrotactile feedback: *Dead Zone* (DZ), *Always On* (AO), or *Sham* (S). Ti (where i goes from 1 to 9 for group 1 and to 11 for group 2) indicates the trial numbers.

Participants stood with their feet together, without shoes, and with their arms hanging at the sides of the body. They wore noise-mask headphones to avoid the influence of disturbances from the vibration sensors and/or environmental noise. The participants were instructed to stand as still as possible with their eyes open or close depending on the trial. They were aware whether or not the vibration was provided in a specific trial. No indication or clue about the informational content of the vibration or the encoding method was provided, but there was a familiarization phase where participants could explore the vibrotactile feedback and understand the encoded information. The experiment was divided in three phases: baseline, familiarization, and test (Figure 3).

#### Baseline

Participants performed a preliminary test, equal for both groups and composed of two trials with a duration of 50 s without the vibrotactile stimulation. In the first trial they had to maintain the standing position with the eyes open (i.e., with the visual feedback: *V*_+_*T*_−_; T1). During this trial they were placed in front of a white wall, at a distance of 1 m, and they had to look at a blue dot target (0.75 cm radius) on the wall. The second trial was performed with the eyes closed (i.e., without the visual feedback: *V*_−_*T*_−_; T2). Between the two trials there was a short pause (about 30 s).

#### Familiarization

The familiarization lasted 30 s. During this phase the participants were free to explore the vibrotactile feedback maintaining the standing position with eyes open or closed, as they preferred. Notice that this allowed the participants to understand that performing correctly the task corresponded to reduce the intensity of the vibration, till a complete silencing only in the DZ method.

#### Test

The first part of the test was composed by three repetitions of two trials with a duration of 50 s each. The first trial was performed without vibrotactile feedback (*V*_−_ *T*_−_; T3-T5-T7), and the second with vibrotactile feedback (T4-T6-T8): *dead zone* method (*V*_−_*T*_+*DZ*_) for group 1 and *always on* method (*V*_−_*T*_+*AO*_) for the group 2.

Participants from group 2 performed also the *sham* test, i.e., they were asked to stand still for three additional 50 s trials where the first and the last trial were without any feedback (*V*_−_*T*_−_; T9-T11), and the second trial with the *sham* feedback (*V*_−_*T*_+*S*_; T10), i.e., a vibrotactile feedback where the vibration intensity was not related to the actual CoM oscillations (see Vibrotactile Feedback Control section). The rationale of testing the effect of *sham* feedback was to verify if measurable sway changes observed in our experiment were due (1) to the informational content of the supplemental vibrotactile feedback or (2) to a mere effect of vibration acting as noise and increasing the perceptive thresholds as in Dhruv et al. (2002), Liu et al. (2002), Janssen et al. (2010), Magalhães and Kohn (2011), Borel and Ribot-Ciscar (2016), and Kwak et al. (2016). In the latter case, we expected that changes—and specifically a reduction—of the postural sway during the exposure to the synchronized informative feedback, would have been maintained during the exposure to the unsynchronized *sham* feedback. This because the *sham* feedback had the same amplitude and frequency of the informative feedback, with the only difference that was unrelated to the actual CoM oscillations. Instead, in the former case, if participants used the information encoded in the vibration in the previous trials, since in the *sham* feedback the vibration would be not related to the actual CoM oscillations, the attempts to use the vibration content would decrease participants' stability, increasing the postural sway.

### Data Analysis

**Figure 4:**
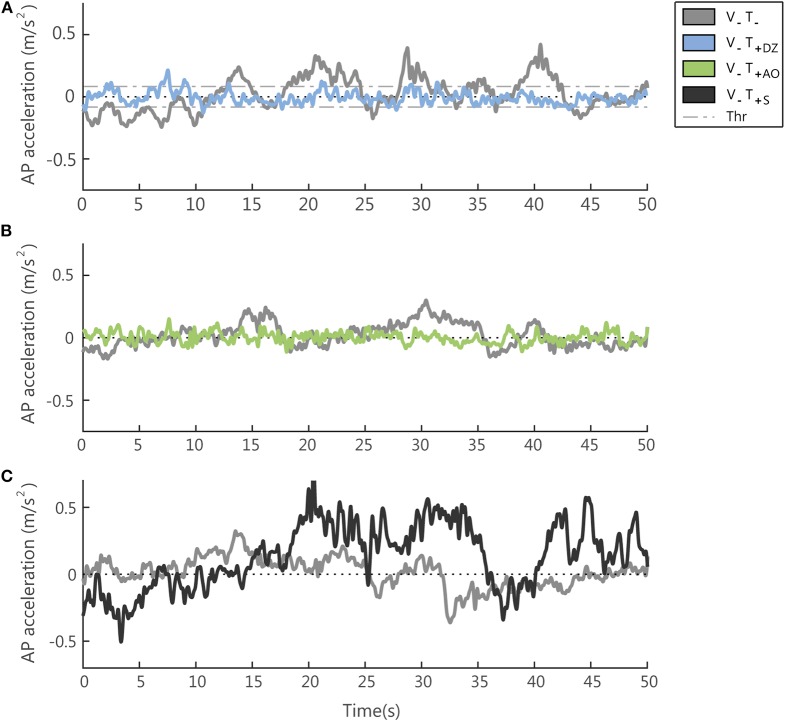
Examples of the accelerometer signal (low-pass filtered, cut off frequency 3.5 Hz) in absence (V_−_T_−_) and presence (V_−_T_+_) of supplemental vibrotactile feedback. Each panel compares, for one typical participant, the accelerometric signal in the (V_−_T_−_) condition with the same signal measured in the three conditions with vibration on: the *dead-zone* method (V_−_T_+DZ_) in **(A)** (note that the dead zone is delimited by the threshold (Thr), i.e., the two dashed lines); the *always on* method (V_−_T_+AO_) in **(B)**; the *sham* feedback (V_−_T_+S_) in **(C)**.

We aimed at investigating the efficacy of synchronized vibrotactile feedback for the reduction of body sway and distinguishing the specific effects of the different encoding methods. The indicators for describing the postural oscillations were extracted from the acceleration signals recorded with the IMU (see Figure 4 for an example) located at L3 level. The accelerometric signal was sampled at a frequency of 50 Hz. During the experiment, for the on line computation of the vibrotactile feedback we used the raw data, while during the off line data analysis to evaluate the postural performance of the participants we took as reference for the signal pre-processing the studies of Mancini et al. (2011, 2012) and Marchesi et al. (2019) and filtered the data with a zero-phase fourth-order Butterworth low-pass (LP) filter with a cut-off frequency of 3.5 Hz. In fact, these studies demonstrated that in quiet standing we can extract reliable indicators of postural stability from the accelerometric signals in the horizontal plane and that these indicators are correlated with the ones extracted from the CoP, both for healthy participants and for people with Parkinson's disease. In other words, according to these studies the higher is the amplitude of these LP filtered signals extracted from the accelerometric signals the greater the postural sway measured by a force platform as shift in the center of pressure. Therefore, in the present study, we referred to an increase/decrease of these signals as an increase/decrease of the postural sway/oscillations.

To evaluate the participants' performance we computed two outcome measures from the acceleration signals (Mancini et al., 2011):

> the Root Mean Square acceleration (RMS), quantifying the magnitude of the acceleration in the spatial-temporal domain;
> the frequency at which the power spectral density reaches the 95th percentile (F95), describing the characteristic of signal in the frequency domain.

We computed both these indicators separately for acceleration components in the anterior-posterior and medial-lateral directions.

#### Statistical Analysis

The baseline data were used (i) to verify that there were no differences between groups before exposure to vibrotactile feedback and (ii) for defining the amplitude of the dead zone (only for the group 2). We also verified that the difference in performance between open and close eyes conditions, expecting a significant worsening in performance when the visual feedback was absent. To do so, we used a repeated measures ANOVA (rm-ANOVA) with one factor within subjects “Visual feedback” (open/close eyes) and one factor between subjects: “Groups” (group 1 vs. group 2).

After that, for verifying if the two methods of encoding the acceleration of the CoM induced changes in the postural sway and if these changes depended on the encoding methods we analyzed the data of the test phase by using a rm-ANOVA with two factors within subjects: “Vibration” (on/off) and “Repetition” (three trials with and three without vibrotactile feedback) and one factor between subjects: “Encoding method” (*dead zone* vs. *always on*). We further investigated significant main and interaction effects by performing a *post-hoc* analysis using Fisher's LSD.

Although we could expect a sizable variability among participants in their baseline performance, we did not normalize the data for the anthropometric parameters or the baseline performance. The reason for this was that in each group the same participant was tested multiple times under different conditions and the rm-ANOVA allowed for individual differences in the baseline, i.e., it allowed testing for the effect of the supplemental feedback (and more specifically for all the factors: vibration on/off, encoding method and repetition) while excluding the influence of different baseline performance across the participants.

Effects were related to repetition in order to highlight (i) learning effects in the vibration trials (ii) after effects in the no vibration trials. Therefore, when the repetition factor or its interactions were significant, we further investigated these results by comparing the first and the last trial on the same condition (presence/absence of vibration). Specifically, in the no vibration condition this was equivalent to test if there were any after effect recorded before exposure to vibrations.

For testing the importance of the informational content encoded in the vibrotactile feedback we compared (three planned comparisons—paired *t*-test), the performance in the *sham* trial with the performance (i) in the last trial with the *always on* method and (ii) in the two trials without vibration before and after the *sham* trial.

The normality of the data was checked with Lilliefors test. The assumption of sphericity necessary to perform rm-ANOVA was verified for all the parameters (Mauchly's test). In all tests the significance level was set at *p* < 0.05. Since we had more than one parameter extracted from the same dataset we verified that all the reported *p*-values—computed without corrections for multiple comparisons—were robust to the Bonferroni-Holm correction (Holm, 1979) and we reported when they were not.

## Results

### Baseline

The first analysis that we performed was to check the performance during the baseline, where the participants had to perform two consecutive trials with (T1) and without (T2) the visual feedback. As expected, we found that all the participants worsened their performance during the closed eyes condition. Specifically, the amplitude of the acceleration signals in the AP and the ML directions significantly increased (RMS: AP: *F*_(1, 22)_ = 36.20, *p* < 0.001; ML: *F*_(1, 22)_ = 22.05, *p* < 0.001). For the F95 parameter there was a significant decrease in the AP direction (*F*_(1, 22)_ = 7.57, *p* = 0.012), which was not found in the ML direction (*F*_(1, 22)_ = 3.69, *p* = 0.068).

The second preliminary analysis was aimed to check that the two groups of participants were equivalent with regards to the baseline performance during unperturbed sway. In particular, we compared the performance in the first two trials, in absence of vibration, and we found no significant differences between the two groups for all the parameters (RMS: AP: *p* = 0.066, ML: *p* = 0.417; F95: AP: *p* = 0.793, ML: *p* = 0.471).

### Supplemental Synchronized Vibrotactile Feedback Reduces the Postural Sway

For investigating the effects of the vibrotactile feedback encoding the CoM information, we analyzed the data collected during the test phase, where participants were required to stand as still as possible with eyes closed and they performed three repetitions of two trials without (T3-T5-T7) and with (T4-T6-T8) supplemental feedback.

#### Encoding Method Effect

We found that for all participants the vibrotactile feedback encoding the accelerometric measurement modified the postural sway, independently of the encoding method (encoding method effect: *p* > 0.42 for all the parameters).

#### AP Direction

When the vibration was applied, in the AP direction, i.e., the direction encoded in the supplemental feedback, there was a significant effect of the vibration on both the RMS and the F95 as displayed in Figure 4 for a typical participant of the group 1 (Figure 4A) and of group 2 (Figure 4B).

**Figure 5:**
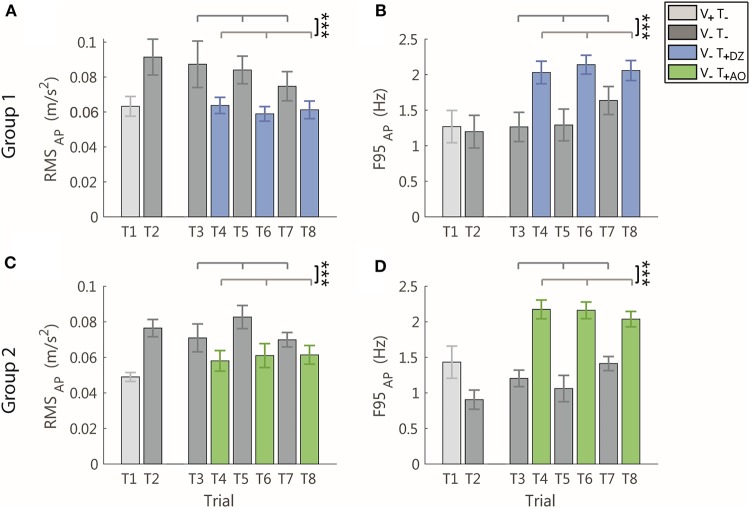
RMS and F95 parameters in the AP direction for group 1 (DZ method) in **(A,B)**, and for group 2 (AO method) in **(C,D)**, respectively. The error-bars represent the standard error of the mean obtained for all the participants. ^*^significant differences of rm-ANOVA: ^***^p < 0.001.

Specifically, the amplitude of the AP acceleration decreased (RMS: *F*_(1, 22)_ = 22.34, *p* < 0.001, Figures 5A,C) and its frequency increased (F95: *F*_(1, 22)_ = 72.02, *p* < 0.001, Figures 5B,D).

#### ML Direction

**Figure 6:**
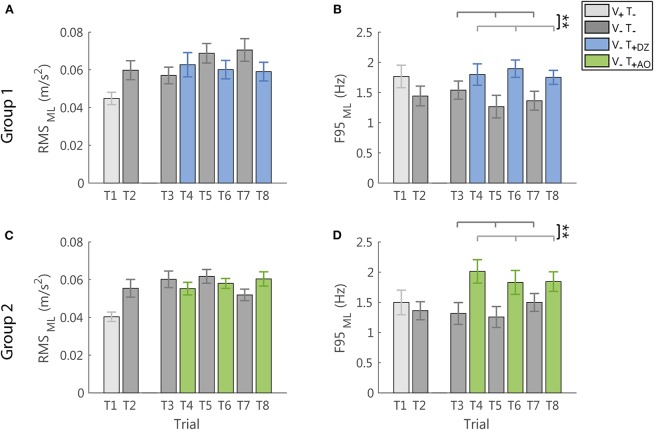
RMS and F95 parameters in the ML direction for group 1 (DZ method) in **(A,B)**, and for group 2 (AO method) in **(C,D)**, respectively. The error-bars represent the standard error of the mean obtained for all the participants. ^*^significant differences of rm-ANOVA: ^**^p < 0.01.

In the ML direction, i.e., the direction not encoded in the supplemental feedback, the vibration produced only a significant increase of the frequency (F95: *F*_(1, 22)_ = 14.17, *p* = 0.001, Figures 6B,D), not followed by a significant change of the amplitude of the accelerometric signal (RMS: *F*_(1, 22)_ = 1.54, *p* = 0.228, Figures 6A,C).

### The *Sham* Feedback Changes the Postural Sway Differently From the Synchronized Feedback

To verify that the reduction of the postural sway above described was effectively due to the information embedded in the feedback related to the accelerometric measurement, we compared the performance in the *sham* trial (T10) with the performance in the last trial with the *always on* feedback method (T8) and the two trials without vibration before (T9) and after (T11) it.

We found that the unsynchronized *sham* feedback determined different changes in the postural sway with respect to the feedback encoding a combination of the actual position and acceleration of the body center of mass in the anterior-posterior direction. The acceleration signals from a representative participant in a trial with the *sham* feedback is reported in Figure 4C.

#### AP Direction

**Figure 7:**
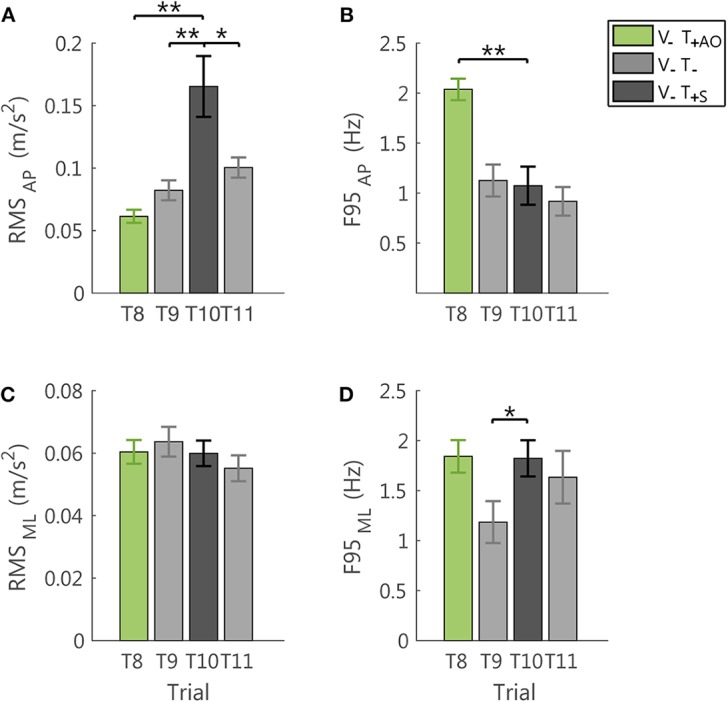
Effects of the *sham* feedback (V_−_T_+S_; T10) in comparison with the performance in the last trial V_−_T_+AO_ (T8) and in the two no vibration trials before and after the *sham* trial (V_−_T_−_; T9, T11). RMS and F95 for the AP direction are reported in **(A,B)**, respectively. RMS and F95 for the ML direction are reported in **(C,D)**, respectively. The error-bars represent the standard error of the mean obtained for all the participants. ^*^significant differences of rm-ANOVA: ^*^p < 0.05, ^**^p < 0.01.

Indeed, the *sham* feedback increased the amplitude of the accelerometric signal in the AP direction, with respect to all the tested conditions, i.e., both the no vibration trials (RMS: T9-T10: *p* = 0.011; T10-T11: *p* = 0.035, the latter was not robust to Bonferroni-Holm correction), and the last trial with AO method (RMS: T8-T10: *p* = 0.002; Figure 7A). For the F95 in the AP direction, the *sham*, differed from the trial with AO method (T8-T10: *p* < 0.001), while no significant differences were observed with respect to the no vibration trials (T9-T10 and T10-T11: *p* > 0.54; Figure 7B).

#### ML Direction

Instead, the F95 of the ML component was higher with respect to the last no vibration trial before the exposure to vibration (T9-T10: *p* = 0.039, not robust to Bonferroni-Holm correction; Figure 7D). For all the other comparisons and the RMS in this direction (Figure 7C), no significant differences were observed (all *p* > 0.34).

### Effects Related to Repetition: Both Synchronized Encoding Methods Determined No Learning Effect, but They Led to Different After Effects

#### Learning Effects in Trials With Vibration

Comparing the trials with the vibrotactile feedback during the test (T4-T6-T8), we found that for both parameters and both groups there were no significant differences among the three repetitions (Fisher's LSD test: all condition *p* > 0.25).

#### After Effects in Trials Without Vibration

In the trials without vibrotactile feedback (T3-T5-T7) the postural sway changed when comparing the performance before (T3) and after (T7) exposure to vibration (T7) and these changes depended on the encoding method.

#### Encoding Method Effect

The amplitude of the acceleration in the ML direction increased for the DZ method, but not for the AO method, which led to a not significant effect of the encoding method factor (interaction effect “Vibration^*^Repetition^*^Encoding method” *F*_(2, 44)_ = 6.23, *p* = 0.004; *post hoc* analysis: V_−_T_−DZ_: T3-T7 *p* < 0.001; V_−_T_−AO_: T3-T7 *p* = 0.093).

In the AP direction, instead, there were no significant after effects for the sway amplitude (no significant interaction “Vibration^*^Repetition^*^Encoding method”: *p* = 0.854), although we observed that the RMS parameter decreased in 8 participants of group 1. We observed after effects also in the frequency domain, where the F95 parameter for the no vibration trials increased across repetitions in the AP direction for DZ method (“Vibration^*^Repetition”: *F*_(2, 44)_ = 11.42, *p* < 0.001, *post hoc* analysis: V_−_T_−DZ_: T3-T7 *p* < 0.001), while the trend was less clear for the AO method, with changes that did not reach a threshold of significance (V_−_T_−AO_: T3-T7 *p* = 0.065).

## Discussion

To investigate the effects of vibrotactile feedback on standing balance, we built a device with two vibration motors, one placed on the back at the L5 level and the other on the correspondent location of the abdomen. The vibration was synchronized with an accelerometric signal encoding a combination of the position and acceleration of the body center of mass in the anterior-posterior direction. We expected that blindfolded healthy participants when exposed to this vibration (1) would modify their postural sway in dependence of the encoding method (AO vs. DZ); (2) the changes depended on the information encoded by the vibration method, i.e., they were not a mere effect of vibration; (3) the vibration did not induce after effects on the natural postural sway in absence of vibration. In short, the results partially matched the expectations: we found that independently from the encoding method, the presence of vibration synchronized with the accelerometric signal decreased the sway amplitude in the AP direction, while increasing its frequency in both directions. The participants accounted for the information encoded in the vibration since the *sham* vibration did not produce the same effects. Surprisingly, we found significant after effects of the vibration for the participants that were exposed to the DZ method.

In the following sections, we discuss in details the results.

### When Exposed to Supplemental Vibrotactile Feedback Synchronized With an Accelerometric Signal Encoding a Combination of the Position and Acceleration of the Body Center of Mass in the Anterior-Posterior Direction, All Participants Modified Their Postural Sways, Independently From the Method Used to Provide This Information

Both encoding methods were able to modify the performance of all participants. Indeed, they reduced the amplitude and increased the frequency of the AP accelerometric signal. These changes can be interpreted as a reduction of the postural sway, i.e., smaller and more frequent postural corrections (Dozza et al., 2005). This effect is consistent with the previous studies, e.g., in Xu et al. (2017) where supplemental vibrotactile feedback was able to modify the postural sway in healthy young participants. The main novelty of these results were that:

1. the changes were mainly on the direction of application of the stimuli, that was also the direction encoded in the supplemental feedback;
2. the presence or absence of a zone without vibration around the natural stance posture had not a specific effect on the postural sway;
3. these changes were obtained by using a simple and low cost device based only on two vibrator motors, while except (Alahakone and Senanayake, 2010), most studies use an array of several vibrator motors.

As for the first result, directional effects on the postural sway were described for the auditory (Dozza et al., 2005) or multimodal (e.g., vibrotactile, auditory and visual (Davis et al., 2010; Huffman et al., 2010) feedback, but to the best of our knowledge similar results were not reported for the vibration feedback with only two motors. Notice that this directional effect could be due to both the information encoded in the vibration or to the positions of the vibration motors that being on the front and the back of the participants could have influenced differently the AP and ML direction, as discussed in the following paragraph.

As for the second, the encoding methods with the idea that participants might attend to the supplemental feedback only outside a certain region of the natural postural sway (Alahakone and Senanayake, 2010) or above a certain threshold of the stimuli. If this is the case, the DZ method would have the advantage to drive the participants' attention to the stimuli only when it is needed could have beneficial effects. The findings that the participants did not have different responses during the exposure of the two encoding methods seems to support this hypothesis.

These results suggest that the proposed simple and low-cost device was able to influence significantly the postural sway, from the initial exposure. Thus independently of the encoding method, the use of the proposed device, were intuitive and effective, i.e., the central nervous system was able to incorporate the supplementary feedback (Janssen et al., 2009) without requiring a long adaptation process. If the informational content was important (see next paragraph), then the process could have been enhanced by the fact that in both cases, the vibrotactile feedback were designed to elicit a repulsive strategy i.e., participants should reduce or silence the vibration intensity for decreasing the postural sway and this method, provided with other more complex matrix of vibration motors, was found to be more effective than that of the attractive strategy (Lee et al., 2012; Kinnaird et al., 2016).

Although these results are interesting and promising, future studies are necessary to verify the effectiveness of this approach. Also in the presence of internal and external perturbations that challenge the balance ability and to verify if different results would be obtained changing the amplitude of the dead zone or how the information of the AP CoM oscillations are encoded in the vibration intensity.

### The *Sham* Feedback Led to Different Sways Patterns Than the Vibrotactile Feedback Encoding a Combination of the Actual Position and Acceleration of the Body Center of Mass in the Anterior-Posterior Direction

The lack of effect on the postural sway of the two different encodings methods described above could be due to the exposure to vibration, with different directional effects because the vibrator motors being located on the front/back of the participants, i.e., the vibration was provided along the AP direction. In fact it is well-known that also a low-level noise vibrotactile stimulation increase the detection of the stimuli, leading to improvements in postural control (Gravelle et al., 2002; Priplata et al., 2002, 2003; Magalhães and Kohn, 2011; Borel and Ribot-Ciscar, 2016; Kwak et al., 2016). To verify whether or not the participants in this experiment integrated their neural control of the informational content encoded in the vibration, we added a trial where the participants of group 2 where exposed to *sham* feedback. In other words, we tested if the modification of the postural sway was the same with unsynchronized feedback with actual postural sway, but with similar amplitude and frequency content. The exposure to the *sham* feedback had different effects than the synchronized informative feedback, determining an increase of the amplitude of the AP direction associated with a decrease of the frequency of the ML direction, with respect to the signal recorded in absence of supplemental feedback. Therefore, our participants when exposed to synchronized informative feedback reduced the amplitude of the AP oscillations and increased their frequency content, by integrating the information encoded in the vibration.

These results are not in contrast with Janssen et al. (2010), where participants with bilateral vestibular loss improved equally with the informative and uninformative vibration. In fact, we specifically tested if our participants accounted for the informational content of vibration when exposed to informative feedback, and the experiment was not designed to verify whether or not informative feedback would lead to the same changes in postural control. In particular, the increased AP acceleration amplitude in presence of the uninformative vibration was probably due not to the mere effect of our *sham* feedback, but to the fact that the participants have learned to integrate in their postural control loop the vibration informational content experienced in the previous trials. Thus, when the feedback become uninformative, its integration on the control loop decreased the postural stability.

This result supports the hypothesis that participants were able to integrate the proposed supplemental feedback in their postural loop control, accounting for its informational content, after a short time from the initial exposure. Thus, this result encourages to further investigate and exploit the possibility of applying this technology and supplemental vibrotactile feedback in long-term training and rehabilitation of postural control abilities.

### The Vibrotactile Feedback Determined Changes on the Natural Postural Sway, Depending on the Encoding Method: the Exposure to DZ Feedback Method Led Short Term After Effects

To investigate the after effects of the exposure to supplemental feedback is important: if present, they modify, either in a positive or negative way, the postural responses of the participants either in the short or in the long term (Goodworth et al., 2009). This could have relevant implications in the sensory substitution domain, e.g., for amputees (Lee et al., 2007), and is a central issue when the technology is used with applications with rehabilitation goals, e.g., in Lindeman et al. (2005) and Asseman et al. (2007). However, the study of after effects of exposure to the vibrotactile feedback has received limited attention (Winstein et al., 1989). Here we made a first step in the direction of investigating this problem, limiting the study to short term effects due to a short exposure to the vibrotactile stimuli. Surprisingly, we found that even a short exposure of few minutes (the entire experiment lasted about 15 min) can induce short term changes in the natural oscillation patterns of the CoM in absence of vibration and these changes depend on the encoding method. Indeed, only the DZ feedback method modified the natural oscillation pattern leading to an increased frequency in the AP direction and, most importantly, to an increased amplitude in of the acceleration component the ML direction. The increase in postural oscillations in the ML direction is usually a negative effect associated to instability. Therefore, this finding needs to be investigated further, extending the study to long-term exposure and to long term after effects of the vibrotactile feedback. As it is, this result seems to suggest that providing feedback method as *always on* instead of one as a *dead zone* is preferable since it allows avoiding undesired after effects.

Notice that based only on the observation of the effect during the exposure to the stimuli we would have concluded that DZ feedback method would be preferable because it reduces the exposure to the stimuli (Dozza et al., 2006). However, the observation of the after effect seems to suggest that the best choice is to keep the vibration *always on* to avoid undesirable effects when the stimuli is turned off. We acknowledge that these are only preliminary results related to the proposed device and protocol. They highlighted the importance to investigate also the after effects of the stimuli, and deeper and larger investigations are needed to drive general conclusions.

### Vibrotactile Synchronized Feedback and Light Touch

In the early 90's it was discovered by Jeka and Lackner (1994) that “fingertip contact influences human postural control”. In particular, it was found that such additional tactile information allowed the subjects to significantly reduce the size of sway movements: very small contact forces, of the order of 1N, could elicit this phenomenon and, at such level of interaction, purely biomechanical explanations would not match the findings while suggesting a multi-sensory integration process, somehow related to the effect investigated in this study. The initial demonstrations mentioned above involved the tandem Romberg standing posture, which is particularly unstable in the frontal plane, however a following study (Clapp and Wing, 1999) obtained similar effects with normal bipedal stance: they also found a positive correlation between the contact force and the reduced oscillation of the CoP in support of the idea of synchronized feedback for the reduction of postural sway. Moreover, it was found that such reduction does not necessarily need to involve the hand but also occurs when different parts of the swaying body (e.g., leg or shoulder) experience a light contact with an environmental referent (Rogers et al., 2001). In any case, it is mandatory that tactile information is not inhibited by any means, such as anaesthetization of the hand (Kouzaki and Masani, 2008). By comparing the effect of different levels of light touch, namely the fact that the stronger the touch the better the sway reduction, it was suggested by Wing et al. (2011) that “heavier contact provides clearer sensory information about sway allowing faster and more accurate compensatory balance adjustments”. In other words, it seems plausible to postulate that the solution adopted by the brain for stabilizing standing upright, in the sense of minimizing as much as possible the unavoidable body sway, is to carry out a multi-sensory data fusion for obtaining the most accurate estimation of the oscillation of the CoM that is essential for closing the stabilization loop. We need to take into account that such critical information is not accessible directly through a specific sensory channel but indirectly through different noisy channels: visual, proprioceptive, and vestibular, in the natural situation. Light touch or synchronized vibrotactile stimulation are artificial channels that can complement the natural ones for improving the accurate evaluation of the CoM sway that is necessary for minimizing its amplitude. There are indeed reasons to believe that sway movements during quiet standing are not noise-driven around a point attractor (the nominal equilibrium posture) but are the results of an intermittent stabilization process attracted by a limit-cycle whose size depends on the inaccuracy of CoM estimation (Bottaro et al., 2005; Asai et al., 2009). From this point of view light touch and vibrotactile synchronized feedback are somehow equivalent. However, the latter one lends itself much more naturally to clinical applications that will be the target of a further development of this study.

### Limitations

We found no difference due to the two encoding methods (AO and DZ) during exposure to the supplemental feedback, thus we added a test with *sham* feedback to verify if the participants took into account the informational content encoded in the vibration. If this were not the case, we would conclude that the lack of difference between the encoding methods were due simply to the fact that participants used the vibration without accounting for the informational content. The results of the test with *sham* feedback allowed us to reject this hypothesis highlighting that the participants previously exposed to the AO feedback method were indeed using the informational content of the vibration. We also expect a similar effect for the DZ method, but this specific test was not included in the present protocol and could be part of a future extension of the research line.

An additional potential effect that was not covered by the protocol used in this study is a “bias effect.” The fact that participants were exposed to *sham* feedback after being exposed to the informative feedback might create a bias: the increased oscillations observed in presence of *sham* feedback were due to the previous exposure to the informative feedback since participants were trying to use the vibration information also during exposure to the *sham* feedback. To verify the effects of this *sham* feedback *per se*, we should have added a group that would have being exposed only to *sham* feedback (or at least exposed first to the *sham* feedback). Pursuing this extension of the line of research performed by the current study we should also have taken into account that the relation between the body sway and the intensity of vibratory noise has a U-like shape, thus only specific levels of noise might induce a decrease of postural performance (Magalhães and Kohn, 2011; Borel and Ribot-Ciscar, 2016; Kwak et al., 2016). However, this was not our goal, but we just wanted to verify the mechanisms underlying the changes in the postural sway due to the vibration we provided encoding the CoM information, as in Krueger et al. for the upper limb supplemental feedback (Krueger et al., 2017).

Finally, with the proposed paradigm, alternating short trials with and without vibration, we specifically aimed at verifying if participants accounted for the vibrotactile feedback we provided in a short time frame (i.e., trial of 50 s) and if that short exposure could determine any modification of the natural oscillation patterns observed before the exposure. Notice that the participants were aware that in the “no vibration trials” the vibration was off. The short exposure to only one of our feedback modalities determined after effects and we believe that, while different protocols could lead to different after effects, their existence was not due to our protocol. However, this point should be further verified in future studies and we acknowledge that the paradigm we choose could have influenced the learning and the related after effects i.e., a different paradigm could have led to different results.

## Conclusion

We developed an easy–to-use, low-cost and portable device that provides the user with supplemental vibrotactile feedback, encoding the position and the acceleration of the CoM in the AP direction. First, we investigated whether the vibrotactile feedback provided by this simple device was able to enhance postural steadiness, finding that the presence of vibrotactile feedback synchronized with the postural sway reduced the amplitude of the sway in the AP direction during the exposure to vibration. The results also highlighted that this reduction did not depend on the time profile of the vibrotactile stimulation, i.e., both a continuous stimulation (AO method) and a paradigm including a dead zone around the natural stance posture (DZ method) determined this effect. However, they had different after effects to the exposure to the vibration: only the DZ method produced short term after effects, increasing the amplitude in the ML and the frequency in the AP direction of recorded signals. Finally, we verified that the reduction during exposure to the supplemental feedback depended—at least for the AO method that was tested with *sham* feedback—on the informational content of the feedback i.e., it was not a mere effect of the vibration since an unsynchronized feedback led to different results.

In conclusion, these results provide new insights about the underling mechanism of the integration of supplemental vibrotactile feedback for balance control. Apart from the physiological interest *per se* about the efficacy of integrating an artificial feedback channel for improving balance, there might also be clinical and epidemiological application for our device and results: evaluating and decreasing the risk of falls in elderly and/or motor impaired participants, and supporting the balance of people wearing exoskeletons or lower limb prostheses.

## Data Availability Statement

The datasets used and/or analyzed during the current study are available from the corresponding author on reasonable request.

## Ethics Statement

The studies involving human participants were reviewed and approved by 222REG2017. The patients/participants provided their written informed consent to participate in this study.

## Author Contributions

GB, VF, and GC built the device. GB and VF collected the data. GB, VF, MC, and AC analyzed the data. All the authors contributed to the writing of the manuscript and to designing the protocol and the device.

### Conflict of Interest

The authors declare that the research was conducted in the absence of any commercial or financial relationships that could be construed as a potential conflict of interest.
